# Intraparenchymal hematoma as a late complication of retrograde intrarenal surgery

**DOI:** 10.1590/S1677-5538.IBJU.2016.0121

**Published:** 2017

**Authors:** Sedat Yahsi, Senol Tonyali, Cavit Ceylan, Kenan Y. Yildiz, Levent Ozdal

**Affiliations:** 1 Turkey Yuksek Ihtisas Training and Research Hospital, Urology Clinic, Ankara, Turkey

**Keywords:** Hematoma, Intrarenal Surgery, RIRS, urolithiasis, computed tomography

## Abstract

A 34 year-old woman was admitted to our hospital with left flank pain. A non-contrast enhanced computerized tomography (NCCT) revealed a 1.5x2cm left proximal ureter stone. Patient was scheduled for ureterorenoscopy (URS) and stone removal. She was submitted to retrograde intrarenal surgery (RIRS). At the postoperative 1st day, the patient began to suffer from left flank pain. A NCCT was taken, which revealed a subcapsular hematoma and perirenal fluid. The patient was managed conservatively with intravenous fluid, antibiotic and non-steroidal anti-inflammatory drug therapy and was discharged at the postoperative 6^th^ day. Two weeks after the discharge the patient was admitted to emergency department with severe left flank pain, palpitation and malaise. KUB (kidney-ureter-bladder) radiography showed double-J stent (DJS) to be repositioned to the proximal ureter. Patient was evaluated with contrast enhanced CT which revealed an 8cm intraparenchymal hematoma/abscess in the middle part of the kidney. A percutaneous drainage catheter was inserted into the collection. The percutaneous drainage catheter and the DJS were removed at the 10^th^ day of second hospitalization. RIRS surgery is an effective and feasible choice for renal stones with high success and acceptable complication rates. However, clinician should be alert to possible complications.

## INTRODUCTION

Retrograde intrarenal surgery (RIRS) refers to the retrograde type of operation for upper urinary tract pathologies by means of an ureteroscope. Since the introduction of first flexible ureteroscopic procedures in 1960’s there have been many refinements in ureteroscopes in terms of image quality, durability and deflection capability (1). With the addition of working channel and irrigation systems to the ureteroscopes and the introduction of holmium: yttrium aluminium garnet (YAG) laser systems, flexible ureteroscopy (FU) has emerged as a choice for both diagnosis and treatment of urinary lithiasis and urothelial malignancies of the upper urinary tract (1, 2).

RIRS is stated as an appropriate alternative to extracorporeal shock wave lithotripsy (ESWL) and percutaneous nephrolithotomy (PNL) for proximal ureter and pyelocaliceal stones smaller than 2cm particularly in obese patients and patients with pyelocaliceal diverticula and infundibular stenosis (2).

We present here a case of intraparenchymal renal hematoma, which occurred three weeks after RIRS.

## CASE REPORT

**Figure 1:**
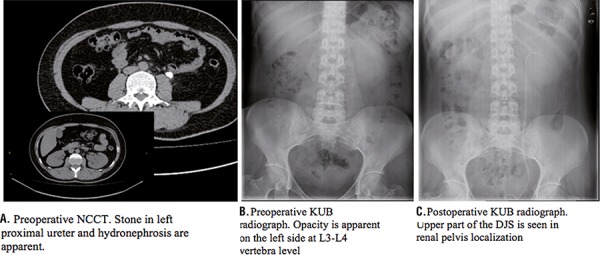
Preoperative KUB radiograph and non-contrast enhanced abdomen CT and postoperative KUB Radiograph.

**Figure 2:**
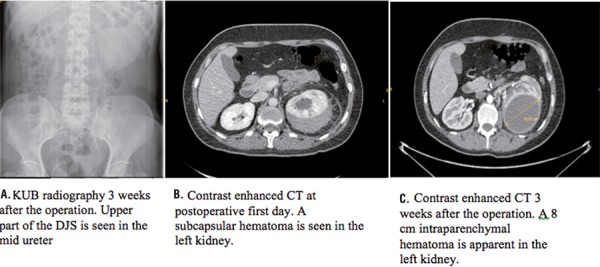
Postoperative KUB radiograph and contrast-enhanced abdomen tomographies.

A 34 year-old woman was admitted to our hospital with left flank pain. A 1.5x2cm left sided opacity was apparent on KUB (kidney-ureter-bladder) radiograph at L3-L4 vertebra level (Figure-1a). The non-contrast enhanced computerized tomography (NCCT) revealed a 1.5x2cm left proximal ureter stone accompanying with grade-2 hydronephrosis (Figure-1b). Patient was scheduled for ureterorenoscopy (URS) and stone removal. The patient had undergone operation within 5 days. During the ureteroscopy with semi-rigid ureterorenoscope the stone migrated into the kidney so we decided to perform a RIRS with flexible ureterorenoscope. A 12F ureteral access sheet (UAS) was passed over a 0.038mm guide-wire up to the ureteropelvic (UPJ) under the guidance of C-arm scope. Then a 270-micron laser fiber was used to fragment the stone in the upper calyx of the kidney. Laser lithotripsy was carried out in 110 minutes with energy of 1.2 joules and a frequency of 8 hertz. Irrigation pressure pump was used to maintain clear vision. Stone fragments larger than 2mm were basketed out with nitinol NGage^TM^ type basket then a 6F 22cm Double-J stent (DJS) was inserted into the ureter. The operation was terminated by insertion of an indwelling urethral catheter. The urethral catheter was removed at postoperative 1st day. Postoperative KUB radiograph demonstrated the DJS to be in the renal pelvis (Figure-1c). At the postoperative 1st day, the patient began to suffer from left flank pain. Whereupon a NCCT was taken which revealed a subcapsular hematoma and perirenal fluid (Figure-2a). Blood pressure and hemoglobin-hematocrit levels were found in normal ranges without a significant alteration compared to preoperative levels, 125/80mmHg and 12.4g/dL-38%, respectively. The patient was managed conservatively with intravenous fluid, antibiotic and non-steroidal anti-inflammatory drug therapy. Repeated ultrasonography examination showed the resolution of the renal hematoma and the patient was discharged at the postoperative 6th day. Two weeks after the discharge the patient was admitted to emergency department with severe left flank pain, palpitation and malaise. Blood pressure was 110/60mmHg and body temperature was 37ºC. Hemoglobin and hematocrit levels were found diminished compared to postoperative levels, 10.7mg/dL and 32%, respectively. Urine culture was negative. KUB radiography showed upper end of the DJS to be fell down from the renal pelvis and migrated to the proximal ureter (Figure-2b). Patient was evaluated with contrast enhanced CT which revealed an 8cm intraparenychemal hematoma/abscess in the middle part of the kidney (Figure-2c). Intravenous antibiotic, analgesic and fluid therapies were given. A percutaneous drainage catheter was inserted into the collection. Output from the drain was 150mL of hemorrhagic fluid at the first day. Culture of this fluid was also negative. Ultrasonography examination on the 7^th^ day confirmed 75-80% shrinkage of the hematoma by the volume with irregular borders. Upon no output from the drain at the following days, the percutaneous drainage catheter and the DJS were removed at the 10^th^ day of hospitalization. The patient had no need of blood transfusion and was discharged at the same day of catheter and DJS removal.

## DISCUSSION AND FUTURE PERSPECTIVES

The main complications of RIRS consist of: fever, flank pain, urinary infection, transient hematuria, acute urinary retention, ureteral and pelvicalyceal abrasion, stone street, subcapsular hematoma, fornix rupture, extravasation, urinoma, ureter avulsion, bleeding requiring transfusion and sepsis (2, 3). Reported complication rates vary between 0% and 25% in different studies (1, 2, 4, 5).

There is no consensus on the use of UAS (1, 6) but it might lessen complication rates by prevention of ureteral injury at repeated FU accesses, maintaining adequate irrigation, decreasing intrarenal pressure and facilitating stone removal (1, 4, 6). On the other hand, it may cause transient ureteral ischemia and ureteral stricture as a late term complication (4, 6).

Physiologic intrarenal pressure is approximately 10mmHg while the threshold for pyelovenous and pyelolymphatic backflow is 30-45mmHg (6, 7). It has been shown that high intrarenal pressure might be a risk factor for septic complications (8).

Hemorrhage can occur as a result of UAS/laser injury or renal calyceal avulsion. Moreover, parenchymal and forniceal rupture because of high intrarenal pressure during the procedure may cause hemorrhage (9, 10). Subcapsular and intrarenal hematomas are rare complications with a few cases in the literature (2). To the best of our knowledge, intraparenchymal hematoma occurring 3 weeks after RIRS following subcapsular hematoma is the first case in the literature. In our case, intraparenchymal hematoma may have arisen due to increased intrarenal pressure after repositioning of DJS.

RIRS surgery is an effective and feasible choice for renal stones with high success and acceptable complication rates. Complications are usually minor that can be treated conservatively. However, clinicians should be alert to possible complications. Shorter operation time, lower stone burden, decrease intrarenal pressure and use of UAS during the procedure may result in reduction of complication rates.
